# A comparative evaluation of the performance of commercially available rapid immunochromatographic tests for the diagnosis of visceral leishmaniasis in Bangladesh

**DOI:** 10.1186/s13071-015-0935-x

**Published:** 2015-06-16

**Authors:** Prakash Ghosh, Md Golam Hasnain, Debashis Ghosh, Faria Hossain, James Baker, Marleen Boelaert, Suman Rijal, Dinesh Mondal

**Affiliations:** Center for Nutrition and Food Security, International Center for Diarrheal Disease Research, Bangladesh, Dhaka Bangladesh; Center for Population Urbanization and Climate Change, International Center for Diarrheal Disease Research, Bangladesh, Dhaka Bangladesh; Department of Public Health, Institute of Tropical Medicine, Antwrep, Belgium; Drugs for Neglected Diseases initiative, New Delhi, India

**Keywords:** Immunochromatographic test, Diagnosis, Visceral leishmaniasis, Bangladesh

## Abstract

**Background:**

Accurate and early diagnosis of Visceral Leishmaniasis (VL) is a prerequisite for proper treatment and restricting disease propagation in enldemic foci. An rK39 antigen-based immunochromatographic test is now recommended for its diagnostic accuracy and operational feasibility at point of care. In endemic regions of Bangladesh, rK39 or rKE16 antigen-based Rapid Diagnostic Tests (RDTs) are routinely performed on whole blood for diagnosis of VL. However, manufacturer’s instructions require use of serum. Therefore, we wanted to assess whether the diagnostic accuracy of these RDTs is as good on whole blood as on serum.

**Methods:**

We evaluated and compared the sensitivity and specificity of five different commercially available RDTs on whole blood and on serum. We enrolled 30 VL patients, 35 endemic healthy controls and 30 Tuberculosis (TB) patients in our study from Mymensingh, a hyper-endemic region in Bangladesh.

**Results:**

The sensitivity of all RDTs ranged between 96.67 % (95 % CI: 82.72-99.44 %) and 100 % (95 % CI: 96.34-100 %). The specificity ranged between 93.85 % (95 % CI: 84.97-98.26 %) and 98.46 % (95 % CI: 91.69-99.74 %), except for the Onsite leishmania Ab (Rev B) kit which showed markedly lower specificity (31.25-58.46 %). There was no significant difference in sensitivity and specificity between blood and serum. The Cohen kappa index (k >0.97) indicated excellent agreement.

**Conclusions:**

We conclude from the study that the use of blood for RDT in lieu of serum is appropriate for diagnosis of VL in peripheral endemic regions provided the manufacturer recommendations are followed and the RDT is of good quality.

## Background

Visceral leishmaniasis or kala-azar is a systemic infection by an obligate intra-cellular parasite, and clinically manifests as chronic fever, fatigue, weakness, loss of appetite and weight loss. Hepatosplenomegaly, anemia, thrombocytopenia and leucopenia are the most common signs. If left untreated, the disease is fatal [1]. The disease is vector-borne, and is poverty–related. In the Indian subcontinent it is caused by *Leishmania donovani*. In Bangladesh, people from 45 out of 64 districts face a potential risk of this fatal disease [2]. Although effective therapies to treat VL are available now, they are potentially toxic and are costly [3]. So, accurate diagnosis before treatment is imperative. In addition to that, visceral leishmaniasis is anthroponotic in the Indian subcontinent and no potential vaccine has been developed against VL till date. So, apart from vector control, early case detection and treatment are essential to restrict disease propagation [3, 4].

Demonstration of the amastigote parasite from spleen aspirates is considered as a gold standard for kala-azar diagnosis for its high sensitivity and specificity. However, spleen aspiration is an invasive method and requires a well-equipped clinical setting to perform [1]. Aspirates from bone marrow and lymph glands are associated with less risk but their sensitivities are not up to the mark [5]. Besides that, several serological and molecular techniques are available for diagnosis of VL [6]. Molecular methods require reference laboratory settings and are not appropriate for first-line health services. Several serological techniques including IFAT, ELISA and DAT have been developed, of which the DAT is most commonly used in field settings for its ease of execution. However, DAT requires multiple pipetting, is time consuming to perform, and its reading requires proper training [6, 7]. Immunochromatographic tests (ICT) are rapid, reliable, simple and feasible to use at point of care, and have been recommended for use in first-line health centres in combination with a clinical case definition [8]. The currently available ICTs are based on rK39 and rKE16 antigen and most of them have to be used on serum samples [9]. The latter is again a constraint for use in a first-line health facility with a very basic laboratory.

According to the Bangladeshi national guideline, an rK39 Rapid Diagnostic Test (RDT) is recommended for VL diagnosis [10]. Notwithstanding manufacturer’s instructions, this RDT is routinely performed on whole blood in peripheral endemic regions of Bangladesh [3]. In addition to that, rK39 dipstick tests have been performed throughout the kala-azar elimination program to do active surveillance and to detect cases early [11, 12]. Few reports have been published on the performance of available commercial RDTs on whole blood compared to serum [3, 5]. It was therefore imperative to evaluate the diagnostic accuracy of the available commercial RDTs. So, the aim of our study was to study the agreement between results obtained in blood and in serum samples of five different types of RDTs; three based on rK39 and two on rKE16 antigen.

## Methods

### Study site

This study was conducted in two sites; the International Centre for Diarrheal Disease Research, Bangladesh (icddr,b) and the field site of parasitology laboratory, icddr,b at Muktagacha, Mymensigh. All participants of this study are permanent residents of Mymensingh, a hyper-endemic region in Bangladesh.

### Study population

The included participants in this study were divided into three groups. The first group consisted of 30 active VL patients without past history of VL. For VL cases our inclusion criteria was either (a) parasitological confirmation or, (b) diagnosis of VL by National guideline along with complete clinical cure assessment after 6 months following treatment [13]. The second group consisted of 35 healthy controls who had no history of VL infection, were more than 18 years old and were negative in the DAT test (titre <1:1600). The third group consisted of 30 TB patients with a positive sputum smear. Patients below 2 years old were excluded from the study.

### Sample collection, transportation and storage

4 ml of blood was drawn from every confirmed VL patient, endemic healthy control and TB patient. Collected sample was divided into two parts and then one was transferred in an EDTA tube and other in an EDTA free tube for separation of serum. After 20–30 min, clotted blood was centrifuged at 2500 rpm for ten min. at room temperature to separate serum. After that, RDT was performed using fresh blood and serum . The remaining blood and serum were stored at −20 °C and transported frozen to the parasitology laboratory at icddr,b for repeating the tests .

### Test kits

Five test kits were selected to perform RDT which fulfilled the operational criteria developed by the VL Laboratory Network. Among five test kits, four were received from two manufacturers, CTK Biotech (Onsite leishmania Ab; Rev A & Rev B rapid test) and Span Diagnostics products (Signal-KA and Crystal-KA) and another from Inbios international Inc. (Kala-azar Detect). After arrival, test kits were unpacked and stored according to the manufacturers’ instructions. Date of arrival, condition of test kits and product lot numbers were carefully recorded. RDT characteristics are listed in Table 1.

**Table 1:** Characteristics of Rapid Diagnostic Tests (RDTs)

Manufacturer	Product	Format	Storage Temp.	Control line	Test line	Sample volume (μl)	Buffer volume (drops)	Min. Reading time (Minute)	Max. Reading time (Minute)
Span diagnostic Ltd. (Surat, India)	Signal®KA	Cassette	2–8 °C	Yes	rkE16	20 μl of serum/blood in 80 μl of saline. 50 μl of diluted sample	Step 1:2	2	10
							Step 2:2		
							Step 3:3		
Span diagnostic Ltd. (Surat, India)	Crystal®KA	Dipstick	2–30 °C	Yes	rkE16	Blood 20 μl	5	15	30
						Serum 20 μl			
CTK Biotech. Inc (San Diego, USA)	On site Leishmania Ab (Rev A) rapid test	Cassette	4–30 °C	Yes	rk39	Blood 40 μl	1	1	15
						Serum 30 μl			
CTK Biotech. Inc (San Diego, USA)	On site Leishmania Ab (Rev B) rapid test	Cassette	4–30 °C	Yes	rk39	Blood 40 μl	1	1	15
						Serum 30 μl			
InBios international, Inc (Seattle, USA)	Kala azar Detect™	Dipstick	4–30 °C	Yes	rk39	Blood 20 μl	3	10	10
						Serum 20 μl			

### Test procedure

Prior to performing the test, RDTs were brought to room temperature and labeled with a random sample code. Each sample was tested with each of the 5 products according to manufacturer’s instructions on serum and on blood. At first, a specific volume of sample was dispensed onto the RDT by micropipette and then chasing buffer was applied drop-wise onto the RDT. The test result was interpreted and then recorded on a standardized form by two technicians at the minimum reading time and not more than 30 min. The second technician was blinded to the first technician’s reading. The presence of both a test and a control line was recorded as positive and the presence of only a control band was recorded as negative. The test result was recorded as invalid if the control band was found to be absent.

### Data analysis

A database was generated in EPI-INFO for data storage and retrieving for further analysis. A double data entry procedure was followed for data entry. Data analysis was performed by SPSS software. The secured preservation of all source documents and electronic records was confirmed until the accomplishment of the study, data analysis and data dissemination. We calculated sensitivity and specificity with 95 % CI using exact binomial methods for proportions. We used Cohen kappa index for agreement testing between test results. The values of Cohen κ coefficients were interpreted according to Landis and Koch; 1.00-0.81: excellent, 0.80-0.61: good, 0.60-0.41: moderate, 0.40-0.21: weak and 0.20-0.00: negligible agreement [9].

### Ethical approval

This study was approved by the icddr,b ethical review committee. Informed written consent was taken from each patient and control subjects or from the legal guardian for minor cases.

## Result

### Participants’ characteristics

The average age was 38 in disease control group, 29 in healthy control group and in VL patient group it was 18. The number of male and female participants was comparable among VL patients, while in disease control group the male–female ratio was 1:9 and in healthy control group it was 8:1. Average spleen size and duration of fever in VL patient group were 5.25 cm and 8 weeks respectively (Table 2).

**Table 2:** Characteristics of the study subjects

Characteristics	VL patients	Healthy control	Controls with TB
	*n* = 30	*n* = 35	*n* = 30
Age (Years)	18.93 ± 13.08	29.09 ± 8.18	38 ± 18.48
*Mean ± SD*	16 (53.3)	0 (0)	3 (11.11)
*Children (<18 years), n, (%)*	14 (46.7)	35 (100)	27 (88.89)
*Adult (>18 years), n, (%)*			
Sex	17 (56.7)	31 (88.6)	17 (56.7)
*Male, n (%)*	13 (43.3)	4 (11.4)	13 (43.3)
*Female, n (%)*			
Spleen size (cm)^a^	5.25 ± 3.98	-	-
*Mean ± SD*			
Duration of fever (Weeks)	8.53 ± 7.92	-	-
*Mean ± SD*			

^a^Spleen size was measured through abdominal palpation and the length was measured from the coastal margin along with its axis

### Diagnostic performance

All RDTs showed excellent sensitivity and specificity (healthy and disease control) for fresh blood and fresh serum with only one exception Onsite leishmania Ab (Rev B) which showed low specificity. Sensitivity of all RDTs for fresh whole blood was found 100 %. For fresh serum, sensitivity was found 100 % of four RDTs except signal KA 96.67 %. Specificity for fresh blood ranged from 58.46 % to 98.46 % and for fresh serum ranged from 55.38 % to 98.46 %. All RDTs showed high sensitivity for stored blood except Rev A and Rev B (Table 3). For stored blood the sensitivity was found 100 % for three RDTs except Rev A and Rev B. Sensitivity for stored serum was found 100 % for four RDTs except kala-azar Detect™ which showed a sensitivity of 96.77 %. Specificity for stored blood was found 98.46 % except Onsite leishmania Ab; Rev A and Onsite leishmania Ab; Rev B and for those tests were invalid. All RDTs gave excellent specificity for stored serum except Rev B. Specificity for stored serum ranged from 100 % to 31.25 % (Table 3 and 4). The high Cohen kappa index (k >0.97) of four RDTs substantiated excellent agreement of test results between fresh whole blood and serum except Onsite leishmania Rev B which showed good agreement (k = 0.69) between fresh whole blood and serum (Tables 5 and 6).

**Table 3:** Product test result (absolute number)

Product	Case (30)											
	Fresh						Stored					
	Blood			Serum			Blood			Serum		
	Positive	Negative	Invalid	Positive	Negative	Invalid	Positive	Negative	Invalid	Positive	Negative	Invalid
*Kalazar Detect*™	30	00	00	30	00	00	30	00	00	30	00	00
*Signal®KA*	30	00	00	29	01	00	30	00	00	30	00	00
*Crystal®KA*	30	00	00	30	00	00	30	00	00	30	00	00
*Onsite leishmania Ab (Rev A)*	30	00	00	30	00	00	-	-	-	30	00	00
*Onsite leishmania Ab (Rev B)*	30	00	00	30	00	00	-	-	-	30	00	00
Product	Healthy Control (35)											
	Fresh						Stored					
	Blood			Serum			Blood			Serum		
	Positive	Negative	Invalid	Positive	Negative	Invalid	Positive	Negative	Invalid	Positive	Negative	Invalid
*Kalazar Detect*™	00	35	00	00	35	00	00	35	00	01	34	00
*Signal®KA*	00	34	01	00	35	00	01	34	00	00	35	00
*Crystal®KA*	00	35	00	00	35	00	00	35	00	00	35	00
*Onsite leishmania Ab (Rev A)*	01	34	00	02	33	00	-	-	-	00	35	00
*Onsite leishmania Ab (Rev B)*	18	17	00	19	16	00	-	-	-	00	35	00
Product	Disease Control (30)											
	Fresh						Stored					
	Blood			Serum			Blood			Serum		
	Positive	Negative	Invalid	Positive	Negative	Invalid	Positive	Negative	Invalid	Positive	Negative	Invalid
*Kalazar Detect*™	02	28	00	02	28	00	01	29	00	01	28	01
*Signal®KA*	02	28	00	02	28	00	01	25	04	01	28	01
*Crystal®KA*	01	29	00	01	29	00	01	29	00	01	28	01
*Onsite leishmania Ab (Rev A)*	02	28	00	02	28	00	-	-	-	03	26	01
*Onsite leishmania Ab (Rev B)*	09	21	00	10	20	00	-	-	-	16	13	01

**Table 4:** Specificity of different products

Product	Fresh	Stored		
	Blood	Serum	Blood	Serum
	Specificity (Healthy Control)			
Kalazar Detect™	100 (96.34–100)	100 (96.34–100)	100 (96.34–100)	97.14 (85.03–95.02)
Signal®KA	100 (89.52–100)	100 (89.90–100)	100 (96.34–100)	100 (89.90–100)
Crystal®KA	100 (96.34–100)	100 (96.34–100)	100 (96.34–100)	100 (89.90–100)
Onsite leishmania Ab (Rev A)	97.14 (85.03–99.52)	94.29 (80.86–99.13)	-	100 (89.90–100)
Onsite leishmania Ab (Rev B)	48.57 (31.39–66)	45.71 (28.84–63.45)	-	20 (8.48–36.95)
	Specificity (Disease Control)			
Kalazar Detect™	93.33 (77.89–98.99)	93.33 (77.89–98.99)	96.67 (82.72–99.44)	96.55 (82.17–99.42)
Signal®KA	93.33 (77.89–98.99)	93.33 (77.89–98.99)	96.15 (80.30–99.36)	96.55 (82.17–99.42)
Crystal®KA	96.67 (82.72–99.44)	96.67 (82.72–99.44)	96.67 (82.72–99.44)	96.55 (82.17–99.42)
Onsite leishmania Ab (Rev A)	93.33 (77.89–98.99)	93.33 (77.89–98.99)	-	89.66 (72.62–97.69)
Onsite leishmania Ab (Rev B)	70 (50.60–85.24)	66.67 (47.19–82.69)	-	44.83 (26.46–64.30)
	Specificity (Healthy and Disease Controls combined)			
Kalazar Detect™	96.92 (89.30–99.54)	96.92 (89.30–99.54)	98.46 (91.69–99.74)	96.88 (89.14–99.53)
Signal®KA	96.88 (89.14–99.53)	96.92 (89.30–99.54)	98.36 (91.17–99.73)	98.44 (91.57–99.74)
Crystal®KA	98.46 (91.69–99.74)	98.46 (91.69–99.74)	98.46 (91.69–99.74)	98.44 (91.57–99.74)
Onsite leishmania Ab (Rev A)	95.38 (87.08–98.98)	93.85 (84.97–98.26)	-	95.31 (86.89–98.97)
Onsite leishmania Ab (Rev B)	58.46 (45.57–70.56)	55.38 (42.53–67.73)	-	31.25 (24.30–57.26)

**Table 5:** Agreement between RDT result on blood versus serum in fresh and stored samples

Blood vs Serum^a^	Agreement index (Cohen’s kappa) with 95 % CI	
	Fresh	Stored
*Kalazar Detect*™	100	95.2 (88.39–100)
*Signal®KA*	97.6 (92.7–100)	100
*Crystal®KA*	100	100
*Onsite leishmania Ab (Rev A)*	97.7 (93.2–100)	-
*Onsite leishmania Ab (Rev B)*	69 (54–84)	-

^a^We didn’t perform RDT on stored blood for Rev A and Rev B following manufacturer’s instruction to avoid performing test on stored blood sample. That is why some kappa values are missing

**Table 6:** Agreement between RDT result on Fresh versus stored in blood and serum samples

Fresh vs Stored*	Agreement index (Cohen’s kappa) with 95 % CI	
	Blood	Serum
*Kalazar Detect*™	97.6 (93–100)	94.99 (88.12–100)
*Signal®KA*	97.6 (92.1–100)	95.2 (88.8–100)
*Crystal®KA*	100	100
*Onsite leishmania Ab (Rev A)*	-	88.4 (78.5–98.3)
*Onsite leishmania Ab (Rev B)*	-	12.7 (0–35.6)

*We didn’t perform RDT on stored blood for Rev A and Rev B following manufacturer’s instruction to avoid performing test on stored blood sample. That is why some kappa values are missing

## Discussion

RDT is routinely performed on blood at primary health centers and district hospitals in endemic areas and it has been considered to be the first-line diagnostic tool for diagnosis of visceral leishmaniasis. This is the first study in Bangladesh which was conducted with a view to make a head to head comparison of the performance of five RDTs when performed on blood and serum. Though this study was not designed to estimate sensitivity and specificity, both rK39 and rKE16 based products except one brand showed excellent sensitivity and specificity for both fresh blood and serum. The sensitivity estimates are of course somehow optimistic, as 20 of the 35 cases were selected on the basis of an RDT according to national guidelines. More importantly, the operational feasibility of every RDT in primary health care was found to be up to the mark. Furthermore, the storing conditions for every RDT were easy to maintain in field settings except the Signal K which requires a storage temperature between 2 °C and 8 °C. Inter-reader variability both in field setting and reference laboratory was minimal. The format (cassette or dipstick) of an RDT is an important factor for the performance of RDT, according to *Rennie et al*. [14]. In this study both formats performed equally well except Rev B and similar findings were reported by *Kumar et al*. [5].

In our study every valid test gave same result for blood and serum, and our results support the findings of *Kumar et al*. [5]. In a previous study disparity was found in several cases. In that study the parasitological status of patients was unknown and few cases gave positive result in RDT when performed on serum but negative on blood. The authors defined the disparity by low antibody titer in corresponding subjects with asymptomatic self-resolving infection [3].

In this study we evaluated five brands of RDT and found good results for fresh blood and serum except one brand. The Onsite Rev B gave lower specificity for both fresh and stored samples. In a previous study, the performance of Rev B was found to be poor in India as well as in Nepal [5]. In addition to Rev B, Rev A also gave invalid result for stored blood samples. According to manufacturer’s instruction, Rev A and Rev B should not be performed on hemolysed blood. As blood stored without cryo-preserver gets hemolysed at freezing temperature. We can attribute this invalid result of Rev A and Rev B to storing procedure. In another multicenter study, RDTs from different companies were evaluated on archived serum samples from Indian subcontinent. Considering the high sensitivity and specificity of Crystal KA, Signal KA and Kala-azar detect, present study results are congruent to the findings of *Cunningham et al*. [9].

For specificity evaluation, RDT was done for endemic healthy controls and TB patients who were considered to be disease controls as VL shows cross-reactions with many diseases, including trypanosomasis, malaria, tuberculosis, leprosy and amebiasis [6]. We found very few positive cases in the disease control group that can be attributed to potential serological cross-reactivity between Leishmania and Mycobacteria [15, 16].

## Conclusion

All findings of this study infer the promising diagnostic accuracy of rK39 and rKE16 based RDTs except Rev B. As all RDTs did not perform equally on archived and fresh blood and serum samples, so manufacturers recommendations should be followed accordingly. In apart from several disparities, the quality performance and excellent agreement substantiate the routine use of fresh blood instead of serum in dipstick test for diagnosis of visceral leishmaniasis in hyper endemic regions of Bangladesh providing RDT should be of good quality.

## Abbreviations

RDT: Rapid diagnostic test
ELISA: Enzyme-linked immunosorbent assay
DAT: Direct agglutination test
VL: Visceral leishmaniasis
IFAT: Indirect fluorescence antibody test
CI: Confidence interval
